# Knowledge and Attitudes Toward Cardiovascular Diseases and Their Risk Factors Among the Najran Population in Saudi Arabia

**DOI:** 10.7759/cureus.46839

**Published:** 2023-10-11

**Authors:** Hamdan AlShehri, Alanoud Alqahtani, Ashjan Al Mansour, Renad Alwadei, Leen Abuanq, Suha Alkhazaim, Abdulmajeed Qashqari, Saleh Al Kulayb

**Affiliations:** 1 Internal Medicine and Cardiology, Najran University, Najran, SAU; 2 Internal Medicine, Najran University, Najran, SAU; 3 Internal Medicine, Taibah University, Madinah, SAU

**Keywords:** saudi arabia, attitude, knowledge, risk factors, cardiovascular disease

## Abstract

Background

Cardiovascular disease (CVD) prevention is of great importance due to the high prevalence of CVDs and elevated treatment expenses among patients and healthcare systems. One of the most effective strategies is the improvement of knowledge and attitude levels toward CVD symptoms and risk factors.

Objective

This study aimed to explore the level of knowledge and attitude about CVD among the general population in Najran city, Saudi Arabia.

Methods

A descriptive cross-sectional study was carried out between November and December 2022 among the Najran population. A structured questionnaire comprised socio-demographic characteristics, attitudes, and knowledge about CVD and risk factors. Ethical approval was taken from the ethical committee.

Results

The study included 527 participants living in Najran city aged 18 to 60 years old. Most participants were Saudi nationals (97.3%, N = 513), two-thirds had a university degree (68.9%, N = 126), and approximately half of them were females (51.8%). Furthermore, about two-thirds of the participants (60.7%, N = 320) showed a good knowledge level, and most reported an excellent attitude (87.3%, N = 460). Participants who had a university degree showed significantly better attitude levels (p-value = 0.043). No factors revealed a significant impact on the knowledge level.

Conclusion

Moderate knowledge and excellent attitude levels were seen among the Saudi population. Increasing the knowledge level among the total population is essential. It will be reflected in their attitude and practice. Thus, structured educational programs and utilization of available CVD guidelines should be strengthened as a better preventive strategy to overcome this condition. Also, using mass and social media to increase population awareness and good health responsibility is an effective way to limit the risk of CVD incidence.

## Introduction

Cardiovascular diseases (CVDs) are one of the leading causes of mortality worldwide [1]. Predictably, the mortality due to those diseases may rise to 23 million by 2030 [2]. CVDs are the major cause of hospital admission, leading to a considerable economic burden on healthcare systems worldwide [3]. For those reasons, knowledge regarding CVD and its associated risk factors are public health necessities that must be considered [4].

Moreover, CVD-related mortality in developing countries is three times more than in developed countries [5]. Regarding Gulf Council Countries (GCC), the prevalence of CVD-related mortality is about 45% of the total death. The prevalence of mortality due to CVDs in Oman and Kuwait was 49% and 46%, respectively. Meanwhile, Saudi Arabia, the United Arab Emirates, Bahrain, and Qatar had less proportion of CVD mortality (42%, 38%, 32%, and 23%, respectively) [6]. Additionally, a systematic review demonstrated that the prevalence of CVD and its associated risk factors are significantly high among the adult population in Gulf countries [7]. Therefore, the Gulf Registry of Acute Coronary Events (Gulf RACE) and the Saudi Project for Assessment of Coronary Events (SPACE) established several projects to recognize the burden of CVD and its associated risk factors for improving the treatment and management of the disease [8].

Unfortunately, several factors are associated with the increased incidence of CVDs. Those risk factors are categorized into non-modifiable and modifiable factors [9]. For instance, non-modifiable factors include sex, age, race, and family history of CVDs [10]. Meanwhile, dyslipidemia, hypertension, diabetes mellitus, unhealthy diet, and smoking are the most common modifiable risk factors for CVDs [11,12] All those factors are the major causes of serious complications such as coronary artery diseases, cerebrovascular diseases, peripheral vascular diseases, and cardiomyopathies [13]. Moreover, numerous studies in Saudi Arabia illustrated that the associated risk factors, especially diabetes mellitus, hypertension, and obesity, increase each year continuously [14,15].

Several studies demonstrated the knowledge and attitudes regarding CVD and its associated risk factors; however, most of those studies were conducted among women [16,17]. To our knowledge, no studies evaluated the knowledge and attitude levels regarding CVD and its related factors in Najran city. Therefore, the current study was conducted to assess the level of knowledge and attitude among the general population in Najran city, Saudi Arabia.

## Materials and methods

The study is a cross-sectional study that was conducted from November to December 2022. Data were collected among the general population in Najran city, Saudi Arabia using an online questionnaire. The participants aged less than 18 years and not from Najran city were excluded from the study. The sample size was calculated using the Raosoft sample size calculator (Raosoft, Inc., Seattle, WA), considering a 50% population proportion, a 90% confidence interval, and a 5% margin of error, and the minimum representative sample was 267 participants. The study got approval from the Research and Ethical Committee of Najran University (approval number: 444-42-35830-DS).

The study questionnaire was adopted from a previous study conducted in Lebanon with some modifications [12]. The questionnaire consisted of 35 questions, all of which were in Arabic via Google Forms (Google, Mountain View, CA). The information in the questionnaire was designed to illustrate socio-demographic characteristics, attitudes, and knowledge toward CVDs and their risk factors.

After data extraction, the data were revised and coded. The statistical calculations were done using the computer program IBM SPSS (IBM Corp, Armonk, NY), release 26 for Microsoft Windows. Data were statistically described in the median (IQR) for continuous data. Frequencies (number of cases) and valid percentages were used for categorical variables.

For calculating the participant’s knowledge level, each correct response was given a score of one point. The total score of knowledge for each participant was calculated by summing their scores in all questions out of 21 points.

Participants’ attitudes were scored on a Likert scale from 0 to 4, according to participants’ answers. The total score for each participant was calculated by summing their scores in all questions out of 32 points. Afterward, the level of knowledge and attitude were categorized into two groups regarding their total score percentage: ≤70 accounted for having a poor level, and >70 accounted for a good level.

Chi-square or Fisher's exact test was performed to compare the associated factors with knowledge and attitude levels. The Mann-Whitney test was conducted to identify the impact of the associated factors on the total knowledge and attitude scores. P-values less than 0.05 were considered statistically significant.

## Results

A total of 527 participants from Najran city filled out the questionnaire and were included in the study. Most participants were from Saudi Arabia (97.3%, N = 513), and two-thirds had a university degree (68.9%, N = 126). Approximately half of them were young adults (aged from 18 to 30 years old; 52.6%, N = 277), married (54.6%, N = 288), and females (51.8%, N = 273). All details are illustrated in Table 1.

**Table 1:** Demographic characteristics of participants The data have been represented as numbers (N) and percentages (%).

Parameters	Category	Count (N = 527)	%
Age	18-30 years	277	52.6
	31-45 years	178	33.8
	46-60 years	65	12.3
	>60 years	7	1.3
Gender	Male	254	48.2
	Female	273	51.8
Marital status	Single	227	43.1
	Married	288	54.6
	Divorced	11	2.1
	Widowed	1	0.2
Educational level	Elementary	13	2.5
	Intermediate	25	4.7
	High school	126	23.9
	University	363	68.9
Nationality	Saudi	513	97.3
	Non-Saudi	14	2.7
Najran city resident	Yes	527	100

A high number of participants recognized most of the CVD risk factors. Despite this, only half of them knew that thyroid dysfunction diseases (49.3%, N = 260) and anemia (53.3%, N = 281) are considered types of CVD risk factors. Furthermore, a relatively high number of participants knew the correct symptoms of CVD, but only 49.0% (N = 258) knew that a considerable increase in weight is an alarm of CVD. The total responses of the participants to the knowledge questions of CVD risk factors and symptoms are described in Tables 2, 3.

**Table 2:** Responses to knowledge questions about risk factors of CVD The data have been represented as numbers (N) and percentages (%).

The knowledge of risk factors causing CVD	Response	Count (N = 527)	%
Smoking	True	497	94.3
	False	15	2.8
	Don’t know	15	2.8
Less physical activity	True	471	89.4
	False	28	5.3
	Don’t know	28	5.3
Fast food	True	498	94.5
	False	16	3.0
	Don’t know	13	2.5
Diabetes mellitus	True	363	68.9
	False	69	13.1
	Don’t know	95	18.0
Hypertension	True	430	81.6
	False	42	8.0
	Don’t know	55	10.4
Hypercholesterolemia and dyslipidemia	True	453	86.0
	False	33	6.3
	Don’t know	41	7.8
Thyroid dysfunction diseases	True	260	49.3
	False	99	18.8
	Don’t know	168	31.9
Anemia	True	281	53.3
	False	110	20.9
	Don’t know	136	25.8
Stroke	True	370	70.2
	False	61	11.6
	Don’t know	96	18.2
Obesity	True	477	90.5
	False	24	4.6
	Don’t know	26	4.9
Alcoholic addiction	True	450	85.4
	False	30	5.7
	Don’t know	47	8.9
History of cardiovascular diseases	True	471	89.4
	False	25	4.7
	Don’t know	31	5.9
Family history of cardiovascular diseases	True	335	63.6
	False	101	19.2
	Don’t know	91	17.3
Increasing age (>55 years in women and >45 years in men) is a CVD risk factor	True	335	63.6
	False	94	17.8
	Don’t know	98	18.6

**Table 3:** Responses to knowledge questions about symptoms of cardiovascular disease (CVD) The data have been represented as numbers (N) and percentages (%).

Symptoms of CVD	Response	Count (N = 527)	%
Pain in the chest, jaw, neck, and left shoulder	True	325	61.7
	False	67	12.7
	Don’t know	135	25.6
Shortness of breath, especially in lying flat	True	434	82.4
	False	36	6.8
	Don’t know	57	10.8
Sweating	True	342	64.9
	False	66	12.5
	Don’t know	119	22.6
Dizziness	True	325	61.7
	False	69	13.1
	Don’t know	133	25.2
Loss of consciousness	True	359	68.1
	False	61	11.6
	Don’t know	107	20.3
Palpitation	True	451	85.6
	False	27	5.1
	Don’t know	49	9.3
Considerable increase in weight	True	258	49.0
	False	103	19.5
	Don’t know	166	31.5

Regarding the attitude toward CVD, the top items showing high attitude levels were eating healthy food with low fats (83.8%, N = 435), increasing physical activities (81.7%, N = 425), and stopping smoking and drinking alcohol (84.0%, N = 435). On the other hand, only 41% (N = 206) of the participants refused to use traditional medicine to avoid CVD. All details are in Table 4.

**Table 4:** Responses to attitude questions about cardiovascular disease (CVD) and risk factors The data have been represented as numbers (N) and percentages (%).

Actions to avoid CVD	Response	Count (N = 527)	%
Eating healthy food with low fats	Strongly agree	435	83.8
	Agree	75	14.5
	Neutral	6	1.2
	Disagree	3	0.6
	Strongly disagree	0	0
Increase physical activities	Strongly agree	425	81.7
	Agree	82	15.8
	Neutral	9	1.7
	Disagree	3	.6
	Strongly disagree	1	.2
Maintain a normal body weight	Strongly agree	407	78.6
	Agree	100	19.3
	Neutral	8	1.5
	Disagree	3	0.6
	Strongly disagree	0	0
Stop smoking and stop drinking alcohol	Strongly agree	435	84.0
	Agree	65	12.5
	Neutral	8	1.5
	Disagree	6	1.2
	Strongly disagree	4	0.8
Always check with the physicians about health state and required medications	Strongly agree	391	75.8
	Agree	100	19.4
	Neutral	19	3.7
	Disagree	6	1.2
	Strongly disagree	0	0
Prefer traditional medicine, such as acupuncture (fine needles inserted in the skin)	Strongly agree	168	33.4
	Agree	61	12.1
	Neutral	68	13.5
	Disagree	120	23.9
	Strongly disagree	86	17.1
Always measure blood pressure, blood sugar, and lipid level	Strongly agree	359	69.3
	Agree	114	22.0
	Neutral	35	6.8
	Disagree	9	1.7
	Strongly disagree	1	0.2
Increase knowledge about CVD through mass media or electronic	Strongly agree	340	67.2
	Agree	93	18.4
	Neutral	42	8.3
	Disagree	22	4.3
	Strongly disagree	9	1.8

**Figure 1:**
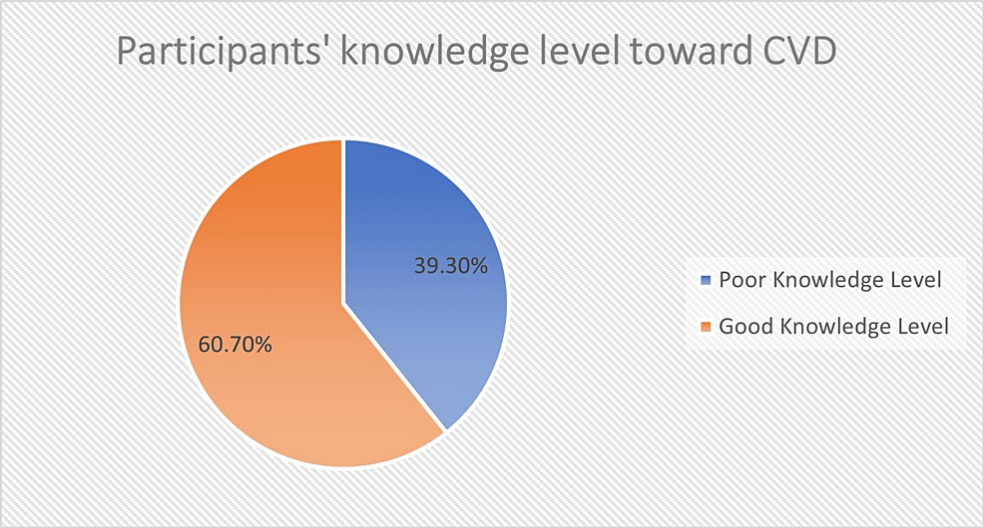
Knowledge level of the participants regarding cardiovascular disease (CVD) The data have been represented as percentages (%).

About two-thirds of the participants (60.7%, N = 320) showed a good knowledge level. Full details are illustrated in Figure 1.

**Figure 2:**
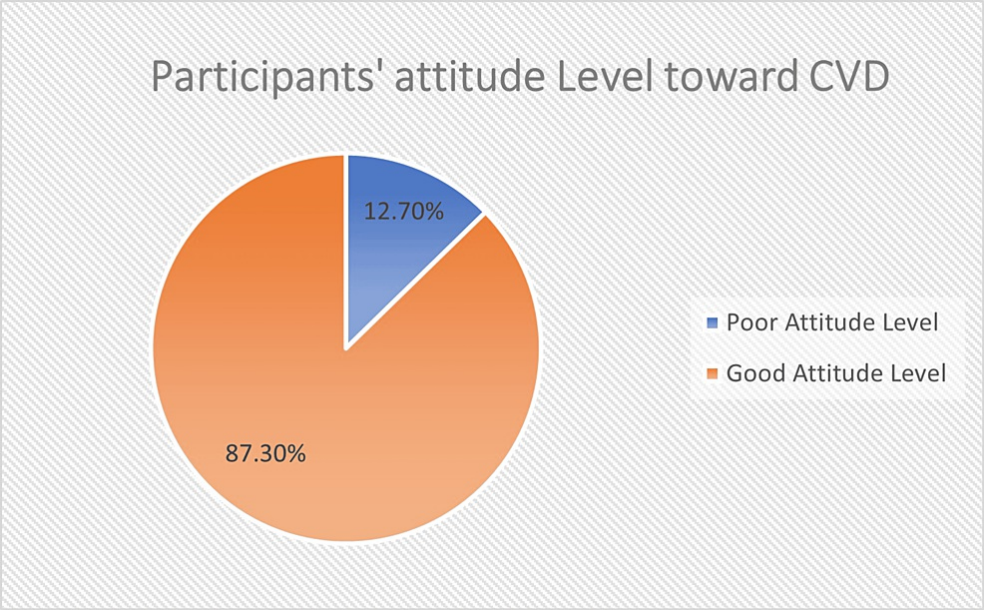
Attitude level of the participants regarding cardiovascular disease (CVD) The data have been represented as percentages (%).

Most participants showed an excellent attitude toward CVD and its risk factors (87.3%, N = 460). All details are in Figure 2.

By comparing the associated factors of CVD with the participants’ levels of knowledge (Table 5), it did not reveal any statistically significant differences regarding the knowledge level.

**Table 5:** Factors affecting the level of knowledge The data have been represented as numbers (percentage) and P-values. P-values less than 0.05 were considered statistically significant.

Factors		Level of knowledge		P-value
		Poor	Good	
Sex	Male	113 (41.4)	160 (58.6)	0.303
	Female	94 (37)	160 (63.0)	
Education	With no university degree	66 (40.2)	98 (59.8)	0.76
	Had a university degree	141 (38.8)	222 (61.2)	
Age	≤45 years	172 (37.8)	37 (51.4)	0.081
	>45 years	35 (48.6)	37 (51.4)	
Marital status	Unmarried	84 (35.1)	155 (64.9)	0.077
	Married	123 (42.7)	156 (57.3)	
Nationality	Saudi	203 (39.6)	310 (60.4)	0.406
	Non-Saudi	4 (28.6)	10 (71.4)	

By comparing the associated factors of CVD with the levels of attitude (Table 6), no statistically significant differences were found except for the education level. Participants who had a university degree showed significantly better attitude levels toward CVD (p-value = 0.043).

**Table 6:** Factors affecting the level of attitude The data have been represented as numbers (percentage) and P-values. P-values less than 0.05 were considered statistically significant. * Statistically significant.

Factors		Level of attitude		P-value
		Poor	Good	
Sex	Male	31 (12.2)	223 (87.8)	0.735
	Female	36 (13.2)	237 (86.8)	
Education	With no university degree	28 (17.1)	136 (82.9)	0.043*
	Had a university degree	39 (10.7)	324 (89.3)	
Age	≤45 years	59 (13)	396 (87)	0.66
	>45 years	8 (11.1)	64 (88.9)	
Marital status	Unmarried	28 (11.7)	211 (88.3)	0.531
	Married	39 (13.5)	249 (86.5)	
Nationality	Saudi	65 (12.7)	448 (87.3)	0.695
	Non-Saudi	2 (14.3)	12 (85.7)	

After comparing the associated factors of CVD with the total score of knowledge (median (IQR) = 17 (6)); factors including sex, education, age, and nationality had no significant impact on the total score. On the other hand, the marital status of the participants significantly affected the total score of the knowledge (p-value = 0.004), where unmarried participants showed higher scores. Full details are described in Table 7.

**Table 7:** Factors affecting the total score of knowledge questions The data have been represented as median, interquartile range, and P-values. P-values less than 0.05 were considered statistically significant. * Statistically significant.

Factors		The total score of knowledge about cardiovascular diseases		P-value
	Categories	Median (17)	Interquartile range (6)	
Sex	Male	16	7	0.465
	Female	16	7	
Education	With no university degree	16	7	0.625
	Had a university degree	16	8	
Age	≤45 years	16	7	0.196
	>45 years	15	9	
Marital status	Unmarried	17	7	0.004*
	Married	15.5	7	
Nationality	Saudi	17	9	0.60
	Non-Saudi	16	7	

In addition, the associated factors were compared with the total score of the attitude (median (IQR) = 28 (3)); factors including sex, education, marital status, and nationality had no significant impact on the total score. Age was a significant factor that significantly impacted the total attitude score, and older participants (aged more than 45 years) showed better attitude scores (p-value = 0.024). Full details are described in Table 8.

**Table 8:** Factors affecting the total score of attitude questions The data have been represented as median, interquartile range, and P-values. P-values less than 0.05 were considered statistically significant. * Statistically significant.

Factors		The total score of participants’ attitudes toward cardiovascular diseases		P-value
	Categories	Median	Interquartile range	
Sex	Male	28	4	0.303
	Female	28	3	
Education	With no university degree	28	5	0.065
	Had a university degree	28	4	
Age	≤45 years	28	3	0.024*
	>45 years	28	4	
Marital status	Unmarried	28	3	0.369
	Married	28	4	
Nationality	Saudi	28	3	0.406
	Non-Saudi	28.5	3	

## Discussion

CVD is considered the most prevalent cause of death around the world and more than 80% of the total mortality rates caused by CVD occur in the Middle East and low-income countries. However, it is considered a preventable disease as most CVD risk factors can be controllable [18].

In general, basic knowledge evaluation as a part of all health education and promotion projects during formative piloting and evaluation could make relevant programs effective and successful with the target audience [19]. In addition, the role of the individual’s attitude and behavior is significantly effective in the occurrence of any disease [20]. Consequently, the present study aimed to explore the knowledge and attitude levels of CVDs and risk factors among Najran populations in Saudi Arabia.

The study revealed that about two-thirds of the participants had a good knowledge of the symptoms and risk factors of CVD. Furthermore, the high knowledge level was translated into an excellent attitude among most participants.

Our results were also consistent with a previous study in Riyadh, Saudi Arabia, which reported that the majority of respondents had a good knowledge level of the risk factors of CVD [21]. In addition, a study in Iran reported a high level of knowledge among adult populations [22]. Another study conducted in Turkey with the same aim as ours showed a moderate knowledge level among the participants [20]. On the other side, Toupchian et al. [23] showed that only about one-third of the respondents had a good knowledge and attitude level.

It was estimated that the level of education is usually an essential factor in any community, which can help make a difference in preventive considerations to control CVDs [24]. In our findings, our respondents’ high level of attitude was associated with their education level. Particularly, the participants with a university degree showed a better attitude than those with less education. On the other hand, education did not impact the knowledge level of the study participants. A similar study in Saudi Arabia supported our results and reported that education had no significant effect on the total knowledge level [24].

In the present study, most participants were aware of the most common symptoms of CVD, including smoking, eating fast food, obesity, hypertension, and hypercholesterolemia. Meanwhile, smoking, obesity, and fast food were the most correctly reported risk factors in this study. Our findings are more like to the results reported in a Saudi survey, which revealed that smoking, obesity, and increased intake of fatty food were the common risk factors identified [25]. Despite these results, it was estimated that smoking and obesity showed high prevalence rates (2.4-52.9% and 35.5%, respectively) among Saudi populations [26]. Thus, the results point out the need for awareness campaigns to fill this gap among the Saudi population and reduce the risk of CVD incidence.

In our findings, half of the participants could not identify anemia as a CVD risk factor. This may be interpreted by the fact that anemia is considered a non-traditional risk factor for CVD [27]. Moreover, many of our participants also did not know that thyroid dysfunction diseases may be one of the CVD risk factors. In fact, thyroid abnormalities may cause alterations in cardiac performance, rhythm, and morphology. However, these cardiac alterations' clinical and prognostic effects are still uncertain [28]. Much improvement is required in some items of general knowledge of CVD risk factors, including thyroid dysfunction diseases and anemia. According to a previous study conducted in Saudi Arabia with different populations, the participants could not identify diet and physical inactivity as risk factors for developing CVD. This could be explained in the light that this study was conducted on a specific group of the Saudi population who are living in Najran city, which could limit results generalization on the whole Saudi population.

Considering that identification of the symptoms of any disease is a golden key for early diagnosis and treatment, recognition of the CVD symptoms was assessed in this study. Most respondents recognized most of the CVD symptoms, such as shortness of breath and palpitations. Meanwhile, weight gain showed a smaller number of responses. A previous study in Kuwait reported that the commonest CVD symptoms identified by respondents were chest pain, pressure, or burning for heart attack followed by sudden numbness or weakness of the face, arm, or leg for stroke [29].

According to the behavioral model, the person with a strong intention motivation, skills, and availability of a conducive environment, there will be more probability that the changes in practice will occur. Thus, a person with a good attitude will have higher motivation to change his or her behavior to practice good things [30]. In this concept, the attitude level of CVD was assessed in this study. The majority of the study participants have shown good attitude levels (87.3%). Meanwhile, they showed poor attitudes regarding performing regular check-ups and learning about CVD through mass media.

In consistence with our findings, an Iranian study has revealed an acceptable attitude level among most of their participants (70%) [22]. Another study performed in Saudi Arabia showed that people have a positive attitude toward the risk factors of CVDs and they believe that they should do regular check-ups. However, they do not maintain a healthy lifestyle [24]. Additionally, another study in Saudi Arabia with different age ranges showed that lower attitude levels were associated with reduced fat and sugar intake and cholesterol level measuring [26].

The limitation of this study is that these results were obtained from the definite sample group of Najran city and cannot be generalized to the entire Saudi population. Meanwhile, it provides directions for healthcare professionals for further studies about CVD knowledge and attitudes with larger sample groups. In addition, the survey depended on self-reported information, which may result in overestimating or underestimating the actual attitude.

## Conclusions

In this study, a moderate percentage of the participants showed good knowledge levels, and higher attitude levels were reported by most of the participants. Increasing the knowledge level among the Najran population is important, as it will be reflected in their attitude and behavior. Thus, providing structured educational programs and utilization of available CVD guidelines should be strengthened as a better preventive strategy to overcome this condition. Also, using mass and social media to increase population awareness and good health responsibility is an effective way to limit the risk of CVD incidence.

These results were obtained from the definite sample group of Najran city and cannot be generalized to the entire Saudi population. Meanwhile, it provides directions for healthcare professionals for further studies about CVD knowledge and attitudes with larger sample groups. In addition, the survey depended on self-reported information, which may result in overestimating or underestimating the actual attitude.
